# Regulation of pathogenic IL-17 responses in collagen-induced arthritis: roles of endogenous interferon-gamma and IL-4

**DOI:** 10.1186/ar2838

**Published:** 2009-10-26

**Authors:** Sujata Sarkar, Laura A Cooney, Peter White, Deborah B Dunlop, Judith Endres, Julie M Jorns, Matthew J Wasco, David A Fox

**Affiliations:** 1 Section of Rheumatology, Department of Medicine, University of Arizona, 1501 N. Campbell Avenue, Rm 6310, Tucson, Arizona 85724, USA; 2 Division of Rheumatology, Department of Internal Medicine, and Rheumatic Disease Core Center, University of Michigan, 1500 E Medical Center Drive, 3918 Taubman Center, SPC 5358, Ann Arbor, Michigan 48109, USA; 3 Department of Pathology, University of Michigan, 1301 Catherine, 5240 Medical Science 1, Ann Arbor, Michigan 48109, USA

## Abstract

**Introduction:**

Interleukin (IL)-17 plays an important role in the pathogenesis of rheumatoid arthritis and the mouse model collagen-induced arthritis (CIA). Interferon(IFN)-γ and IL-4 have been shown to suppress Th17 development *in vitro*, but their potential immunoregulatory roles *in vivo *are uncertain. The goals of this study were to determine the relationship between Th17 responses and disease severity in CIA and to assess regulation of IL-17 by endogenous IFN-γ and IL-4.

**Methods:**

DBA1/LacJ mice were immunized with type II collagen in complete Freund's adjuvant (CFA) to induce arthritis, and treated with neutralizing antibody to IFN-γ and/or IL-4. Systemic IL-17, IFN-γ, and IL-4 were measured in serum. At the peak of disease, cytokine production was measured by ELISA of supernatants from spleen, lymph node and paw cultures. Paws were also scored for histologic severity of arthritis.

**Results:**

Joint inflammation was associated with a higher ratio of systemic IL-17/IFN-γ. Neutralization of IFN-γ accelerated the course of CIA and was associated with increased IL-17 levels in the serum and joints. The IFN-γ/IL-4/IL-17 responses in the lymphoid organ were distinct from such responses in the joints. Neutralization of IL-4 led to increased arthritis only in the absence of IFN-γ and was associated with increased bone and cartilage damage without an increase in the levels of IL-17.

**Conclusions:**

IL-4 and IFN-γ both play protective roles in CIA, but through different mechanisms. Our data suggests that the absolute level of IL-17 is not the only determinant of joint inflammation. Instead, the balance of Th1, Th2 and Th17 cytokines control the immune events leading to joint inflammation.

## Introduction

IL-17 has recently been implicated in the pathogenesis of multiple autoimmune diseases, including rheumatoid arthritis (RA) and the mouse model collagen-induced arthritis (CIA). Patients with RA have higher levels of IL-17 in their serum and synovial fluid than normal controls or patients with osteoarthritis (OA) [1-3]. IL-17-producing Th17 cells are present in the T cell-rich areas of RA synovium [4] and induce the expression of receptor activator of NF-kB ligand (RANKL), which aids bone resorption [2,5,6]. Furthermore, high levels of mRNA for IL-17 and TNF-α in the RA synovium are predictive of joint damage progression, while high levels of interferon (IFN)-γ mRNA are predictive of protection from damage [7]. These findings indicate that IL-17 is a key pathogenic cytokine that is relevant to the downstream events associated with autoimmune joint inflammation. In addition, studies that have employed strategies to up-regulate, neutralize or delete IL-17 have shown, quite consistently, that Th17 cells have a pathogenic role in CIA [8-10].

RA and CIA are complex diseases with requirements for systemic and target organ specific T cell and B cell activation, and these processes are positively and negatively regulated by multiple cytokine networks. *In vitro *studies show that Th17 development is down-regulated by IFN-γ and IL-4, cytokines derived from Th1 and Th2 cells, respectively [11,12].

The role of IFN-γ in animal models of arthritis is complex, with evidence for both protective and pathogenic functions. Previous studies have found that mice deficient in either IFN-γ or IFN-γ receptor develop more severe CIA than wild type counterparts [13-16]. Proteoglycan-induced arthritis, on the other hand, is dependent on IFN-γ and independent of IL-17 [17,18]. IFN-γ clearly has the ability to induce inflammation in some settings, but it can also inhibit Th17 differentiation and thereby reduce inflammation. The net effect of IFN-γ may depend on the phase of disease and the location - such as the joint versus the spleen or lymph node. By administering neutralizing antibodies at different time points, one study suggested that IFN-γ has pathogenic effects in the early phase of disease but protective effects in the later stages [19]. Although this study did not measure IL-17, one plausible interpretation of these results is that IFN-γ possibly takes on a protective role after Th17 cells become overabundant and highly pathogenic.

Similar to IFN-γ, evidence for the role of IL-4 in arthritis is complex. IL-4-based interventions can prevent or alleviate joint inflammation and bone damage in multiple animal models of arthritis [20-22]. We have shown previously that systemic injection of dendritic cells genetically engineered to produce IL-4 (IL-4 DCs) attenuates CIA [21]. Further mechanistic studies revealed that IL-4 secreted from IL-4 DCs is a potent suppressor of IL-17 production by T cells from the early phase of CIA [23]. These results suggest that endogenous IL-4 could also play a protective role in arthritis by suppressing IL-17 in the early phase of disease. However, it leaves open the possibility that it could also have pathogenic effects by suppressing production of IFN-γ, once IFN-γ has taken on a protective role. In addition, IL-4 reduces bone damage in established CIA, and is necessary for the development of arthritis, possibly due to the important role of IL-4 in B cell activation and antibody production [20,24]. Thus, like IFN-γ, IL-4 may have both protective and pathogenic roles in CIA, depending on the stage of disease, location of IL-4 production and relative abundance of other cytokines.

This suggests that *in vivo *the balance of IFN-γ, IL-4 and IL-17 is important in the pathogenesis of CIA. The experiments described in the current paper were designed to further test this hypothesis, in a CIA model in which not all immunized mice develop clinical arthritis. We measured systemic cytokine levels at several time points, as well as *in vitro *cytokine production from lymphoid organs and joints during the peak of disease. Our data shows that disease correlates with the systemic IL-17/IFN-γ ratio rather than the absolute level of any single cytokine. Administration of neutralizing antibodies to IFN-γ and/or IL-4 differentially altered the cytokine responses and course of disease.

## Materials and methods

### Mice

Male 8- to 10-week-old DBA1 mice (Jackson laboratories, Bar Harbor, Maine, USA) were housed in specific pathogen free conditions. All procedures were approved by the University Committee for the Use and Care of Animals of the University of Michigan.

### Collagen-induced arthritis

Complete Freund's adjuvant (CFA) was prepared by mixing heat inactivated mycobacterial strain H37Ra in incomplete Freund's adjuvant at 4 mg/ml. Lyophilized chicken collagen (Chondrex, Redmond, WA, USA) was dissolved overnight in acetic acid at 4 mg/ml. CFA and collagen were mixed at 1:1 to form an emulsion. 100 μg of collagen was injected intradermally at the base of the tail. Mice were scored for arthritis every other day from day 15 after immunization.

Scoring was performed as follows: 0 = no swelling or redness of paws or digits; 1 = swelling and redness in one to two digits; 2 = swelling and redness over ankle or three or more digits or midfoot; 3 = swelling and redness over ankle and midfoot or digits and midfoot; 4 = swelling and redness over entire foot or ankylosis.

### Neutralizing antibody protocol

For these experiments, neutralizing rat antibodies to mouse IFN-γ (clone R46A2) or IL-4 (clone 11B11) were purified from hybridomas (ATCC, Manassas, VA, USA) and used at 100 μg/mouse/per day. Neutralizing antibody to IL-17 (clone M210) was a kind gift from Amgen (Thousand Oaks, CA, USA). The antibodies were injected intraperitoneally from day 10 to 20. Rat IgG at 100 μg/mouse/day was used as a control.

### Tissue collection and assays

Mice were sacrificed by CO_2 _inhalation. Blood was collected by cardiac puncture into serum separator tubes, and serum was frozen at -80°C for cytokine assays to be performed at a later date. For some assays 100 μl of blood was collected serially from tail bleeds on days 0, 14, 28 and 42. Spleens and inguinal lymph nodes were collected and single cell suspensions of these tissues were made and used in re-stimulation assays to assess antigen-specific responses. Re-stimulation was performed by culturing single cell suspensions of spleens or lymph nodes for five days with 100 μg/ml of chicken collagen. Supernatants were collected at day 5 of culture and analyzed for various cytokines. Paws were collected by incising at the fur line. The paws were cut up into small pieces and cultured in medium overnight at 37°C. Supernatants were collected for various cytokine assays.

### ELISA

IFN-γ, IL-4 and IL-17 were measured by ELISA. Plates were coated with anti-IFN-γ antibody (clone R46A2 or XMG1.2, Biolegend, San Diego, CA, USA), anti-IL-4 antibody (clone11B11) or anti-IL-17 antibody (clone TC11-18H10.1, Biolegend, San Diego, CA, USA). Plates were blocked and then loaded with tissue culture supernatants or serum. The plates were washed and developed with biotin conjugated detection anti IL-17 antibody (clone TC11-8H4, Biolegend, San Diego, CA, USA), anti-IFN-γ detection antibody (clone XMG1.2 or R4-6A2 Biolegend, San Diego, CA, USA), or anti IL-4 detection antibody (clone BVD6-24G2, BD Pharmingen, San Jose, CA, USA) and streptavidin horseradish peroxidase followed by tetramethylbenzidine (TMB). Colorimetric intensity was then quantitated in a Biorad (Hercules, CA, USA) ELISA plate reader using KC4 software (Biotek, Winooski, VT, USA). IL-10 ELISA was performed using a kit from BD Pharmingen (San Jose, CA, USA), following the manufacturer's protocol.

### Flow cytometry

Splenocytes or single cell suspensions of draining inguinal lymph nodes were cultured with collagen overnight and stained with fluorescent labeled anti-IL-17 (clone TC11-18H10.1, Biolegend (San Diego, CA, USA), anti-CD4 (clone GK1.5, Biolegend, San Diego, CA, USA), anti-IFN-γ (clone XMG 1.2, Biolegend, San Diego, CA, USA), and anti-IL-4 (clone 11B11, Biolegend, San Diego, CA, USA) antibodies following six-hour stimulation with phorbol 12-myristate 13-acetate (PMA)/ionomycin/BrefeldinA. The cells were then analyzed in FACS Calibur and data analyzed using Cell Quest software (BD, San Jose, CA, USA).

### Histologic scoring

Mouse hind paws were used for histology scoring. The paraffin-embedded tissue was sectioned in an axis longitudinal to the tibia. Three sections from the center of each paw were stained with H&E and scored by two independent blinded observers. Inflammatory infiltrate, synovitis (synovial hyperplasia), cartilage destruction and bone involvement were each scored on a scale of 0 to 3. 0 = no change, 1 = mild, 2 = moderate and 3 = severe.

### Statistical analysis

Serum cytokine analysis was performed for each mouse in triplicate. For some experiments the ELISA data for an entire group were pooled and expressed as mean +/- standard deviation. ELISA assays on culture supernatants were performed in triplicate. Data are presented as mean +/- standard error of the mean. Significance was analyzed by using the Student's t-test or analysis of the variance.

## Results

### Imbalance of systemic IL-17 versus IFN-γ (IL-17/IFN-γ) is associated with joint inflammation

Previous reports suggest that susceptibility to arthritis in various mouse strains correlates with high levels of IL-17 and low levels of IFN-γ [25,26]. In the CIA model, DBA/1 mice given a single intradermal injection of type II collagen in CFA develop a non-synchronous arthritis beginning at around day 20 with highly variable severity. Without booster immunization, as many as 40% of the mice do not develop clinical arthritis by day 45. As Th17 responses have been associated with autoimmunity and CIA, we measured serial serum IL-17 levels after collagen immunization, with the hypothesis that mice with a more robust systemic Th17 response develop arthritis, while mice with a weaker Th17 response do not develop arthritis. We also measured serum IFN-γ levels over time to determine if mice with weak Th1 responses developed more arthritis than those with robust Th1 responses. Mice were immunized on day 0 and serum was collected on days 0, 14, 28 and 42 for measurement of cytokines by ELISA.

**Figure 1:**
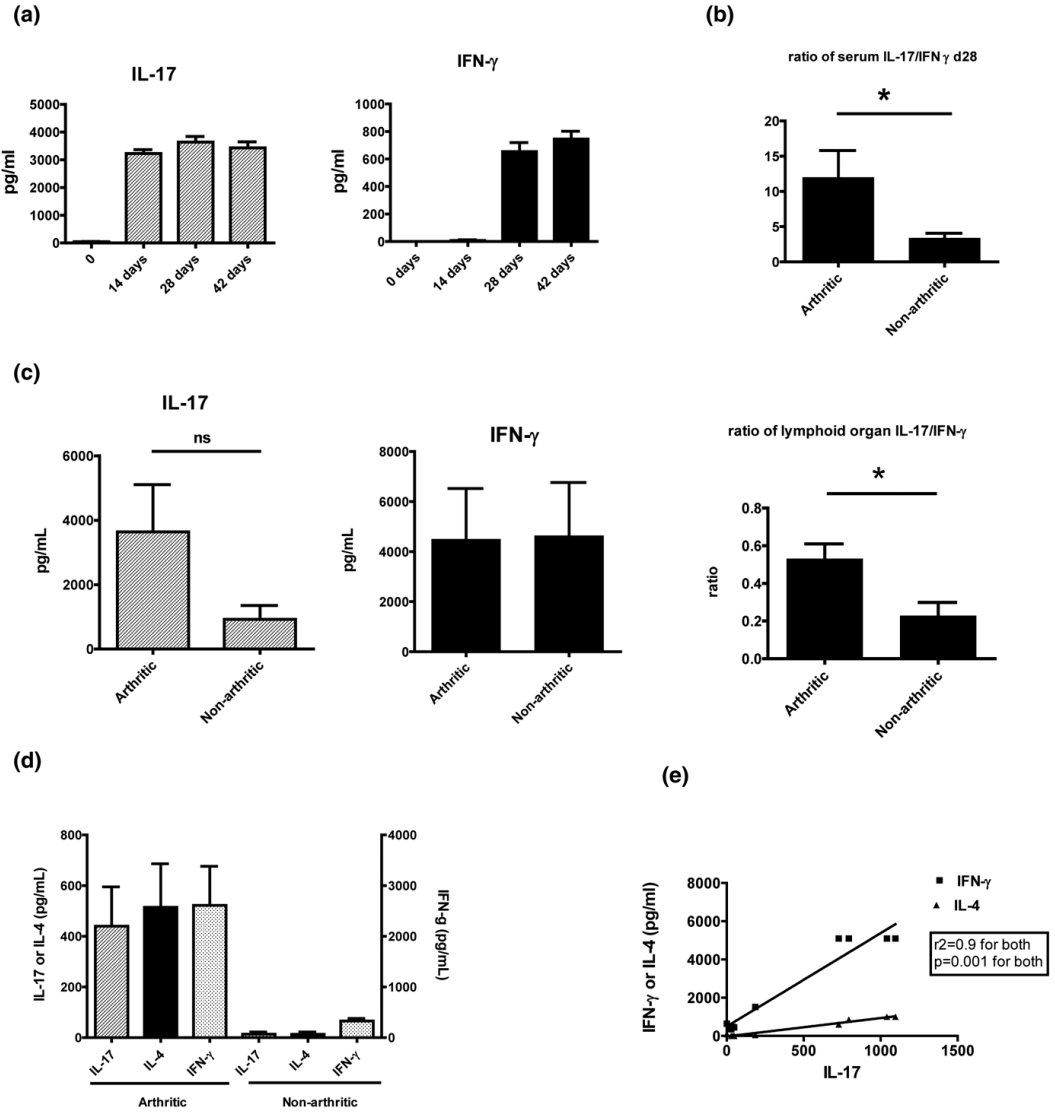
Systemic and synovial IL-17 responses in collagen-induced arthritis. **(a) **Male DBA mice were immunized with chicken type II collagen and complete Freund's adjuvant (CFA) on day 0. Serum was collected serially by tail bleeds on days 0, 14, 28 and 42. IL-17 and IFN-γ were measured by ELISA in triplicate. Data is presented as composite mean of cytokine levels from 30 mice from two experiments. **(b) **Arthritis was assessed by clinical scoring of paw swelling and redness every other day from day 20. Mice with a clinical score of two in at least one paw were considered arthritic. IL-17/IFN-γ is the ratio of the absolute level of IL-17 in serum to the absolute level of IFN-γ in serum at day 28 after immunization with collagen and CFA. Data is presented as composite mean of 30 mice from two experiments. * mean significant with *P *= 0.01 and variance significant with *P *< 0.001. **(c) **Splenocytes and single cell suspension of draining inguinal lymph nodes, from day 28 after collagen and CFA immunization, were cultured for five days in the presence of collagen rechallenge. Supernatants were analyzed for IL-17 and IFN-γ by ELISA. Arthritis was scored as in materials and methods. Mice with a score of at least two in one paw were considered arthritic. Data is presented as composite mean of 30 mice from two experiments. * *P *< 0.05, ns = not significant. **(d) **Paws were collected from mice 28 to 32 days after collagen immunization. Both front and hind paws were cut into small pieces and cultured overnight in media. Supernatants were collected for measurement of IL-17, IL-4 and IFN-γ by ELISA. Data is presented as composite mean of 20 mice from two experiments. **(e) **Correlation analysis of paw IL-17 to paw IFN-γ and IL-4. Data is from arthritic paws. Non-arthritic paws did not have measurable quantities of IL-17.

Figure 1a shows that serum IL-17 was markedly elevated by day 14 and remained elevated until at least day 42. All mice developed long-lasting elevation of serum IL-17, whether or not they developed arthritis. Serum IFN-γ was notably elevated by day 28, later than the earliest measured elevations of IL-17, and there was a wide range in its absolute level. IL-4 was not detectable in serum at any time point (data not shown). In order to assess disease outcome with a composite measurement of Th17 and Th1 responses, the ratios of IL-17 to IFN-γ concentrations were calculated. Figure 1b shows that mice that developed arthritis had a significantly higher ratio of serum IL-17/IFN-γ on day 28.

To quantify antigen-specific Th1 and Th17 responses in mice with or without arthritis on day 28, spleen and draining lymph node cells were re-stimulated *in vitro *with collagen, and IFN-γ and IL-17 were measured in the supernatant by ELISA (Figure 1c). IL-4 was not detectable (data not shown). Although serum IL-17 levels were fairly uniform among arthritic and non-arthritic mice, the collagen-specific Th17 responses in the lymphoid organs were much more variable and there was a trend towards increased IL-17 in arthritic mice (although it did not reach statistical significance). Consistent with the cytokine ratios observed in serum, however, arthritic mice had a significantly higher IL-17/IFN-γ ratio in culture supernatants than non-arthritic mice.

These results suggest that both Th1 and Th17 responses are initiated after administration of collagen and CFA, and that disease progression depends on the balance between the two competing lineages rather than the absolute strength of either alone. We next examined whether T cell responses in the target organ correlated with clinical disease scores. IL-17, IFN-γ, and IL-4 were measured in paws by mincing and culturing them overnight, without exogenous antigen, followed by ELISA of the supernatants (Figure 1d). The Th17 response in the joint was distinct from the systemic response, in that only paws from arthritic mice produced IL-17. Interestingly, non-arthritic mice did not have any measurable IL-17 in the paw cultures even though they had similar serum levels of IL-17 compared with arthritic mice. Furthermore, IL-4 and IFN-γ, were also only detectable in cultures of arthritic paws, and the amounts produced correlated positively with the amount of IL-17 produced (Figures 1d and 1e). These results suggest that inflammatory responses in the secondary lymphoid tissues may be distinct from those in the target organ. This is consistent with a recently published study reporting that Th17 cells are the major source of IL-17 in the lymph nodes whereas γδ T cells are the major producers of IL-17 in the inflamed joints [27].

### Regulation of IL-17 responses by the Th1 cytokine IFN-γ during the initiation phase of arthritis

**Figure 2:**
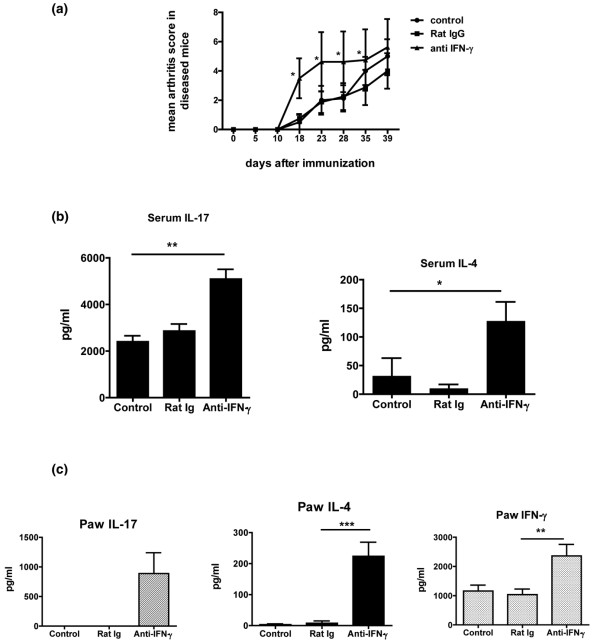
Regulation of IL-17 responses by the Th1 cytokine IFN-γ during the initiation phase of arthritis. **(a) **Neutralizing antibody to IFN-γ (clone R46A2, 100 ug/mouse/day) was administered intraperitoneally from day 10 to 20 after immunization with collagen and complete Freund's adjuvant (CFA). Rat IgG was used as isotype control. The control group did not receive any antibody. Arthritis was assessed from day 10 by clinical scoring. Data is representative of two experiments, with n = 7 in each group in each experiment. * *P *< 0.05. **(b) **Serum from day 21 to 23 after collagen immunization was analyzed for IL-17 and IL-4 by ELISA. Data is presented as mean +/- standard error of the mean (SEM). Data is representative of two experiments, with n = 7 in each group. * *P *< 0.05 and ** *P *< 0.001. **(c) **The paws of mice from the different groups were cultured in media overnight. The supernatants were analyzed for IL-17, IL-4, and IFN-γ by ELISA. Data presented as mean +/- SEM. Data is representative of two experiments, with n = 7 in each group. *** *P *< 0.0001. ** *P *< 0.01.

As the absolute level of IL-17 was not predictive of arthritis, but the balance of endogenous IL-17 and IFN-γ appeared to be important, we chose to perturb this balance by neutralizing endogenous IFN-γ. Regulation of IL-17 responses during the early phase of arthritis is critical, because serum IL-17 is detectable by day 14 (Figure 1a) and [28] IL-4 secreting DCs administered on day 14 attenuate CIA and suppress *in vitro *production of IL-17 by T cells [21,23]. Therefore, neutralizing antibodies to IFN-γ were administered from day 10 to 20 after collagen immunization, targeting this early initiation phase of CIA. As shown in Figure 2a, this resulted in an accelerated course of arthritis. However, the incidence and severity at day 40 was the same amongst the different groups. This result is consistent with previously reported studies [24], and suggests that IFN-γ has a protective role in the early response to immunization with collagen and CFA.

In the absence of endogenous IFN-γ, arthritis peaked by day 20, at which point the severity was significantly different from the control groups. Hence we chose to evaluate systemic and articular immune events around day 20. Serum from day 21 to 23 after collagen immunization was analyzed for IL-17 and IL-4 (Figure 2b). Mice that received neutralizing antibody to IFN-γ had higher levels of IL-17 and IL-4. Although high levels of IL-17 and IL-4 were measured in the serum from these mice, augmented IL-17 and IL-4 responses were not seen in collagen-stimulated spleen and lymph node cultures [Figure S1 in Additional data file 1]. We also measured IL-17, IL-4, and IFN-γ levels in the inflamed joints by ELISA of supernatants from overnight culture of dissected paws (Figure 2c). Consistent with previous data depicting the differences between the systemic and joint specific Th1/Th2/Th17 responses, increased levels of IL-17, IL-4, and IFN-γ were found in the paws of mice which developed accelerated arthritis after receiving IFN-γ neutralizing antibodies. The mice that did not receive anti-IFN-γ antibody (Rat IgG and Control groups) did not develop arthritis by day 21 to 23 and consequently the levels of IFN-γ, IL-4, and IL-17 in the paws remained significantly lower than the mice that received anti-IFN-γ.

### Role of endogenous IL-4 in regulation of CIA

IL-4 has been shown to suppress IL-17 production *in vitro *during immune responses to collagen [23]. It therefore seemed possible that the increased levels of IL-4 in the absence of IFN-γ might fulfill a regulatory role. However, it has also been suggested that IL-4 might have pathogenic effects during the early phase of arthritis [29,30]. Additional experiments were therefore performed to assess the role of IL-4 in mice that were treated with neutralizing antibody to IFN-γ, by neutralizing both endogenous IFN-γ and IL-4 during the early phase of arthritis.

**Figure 3:**
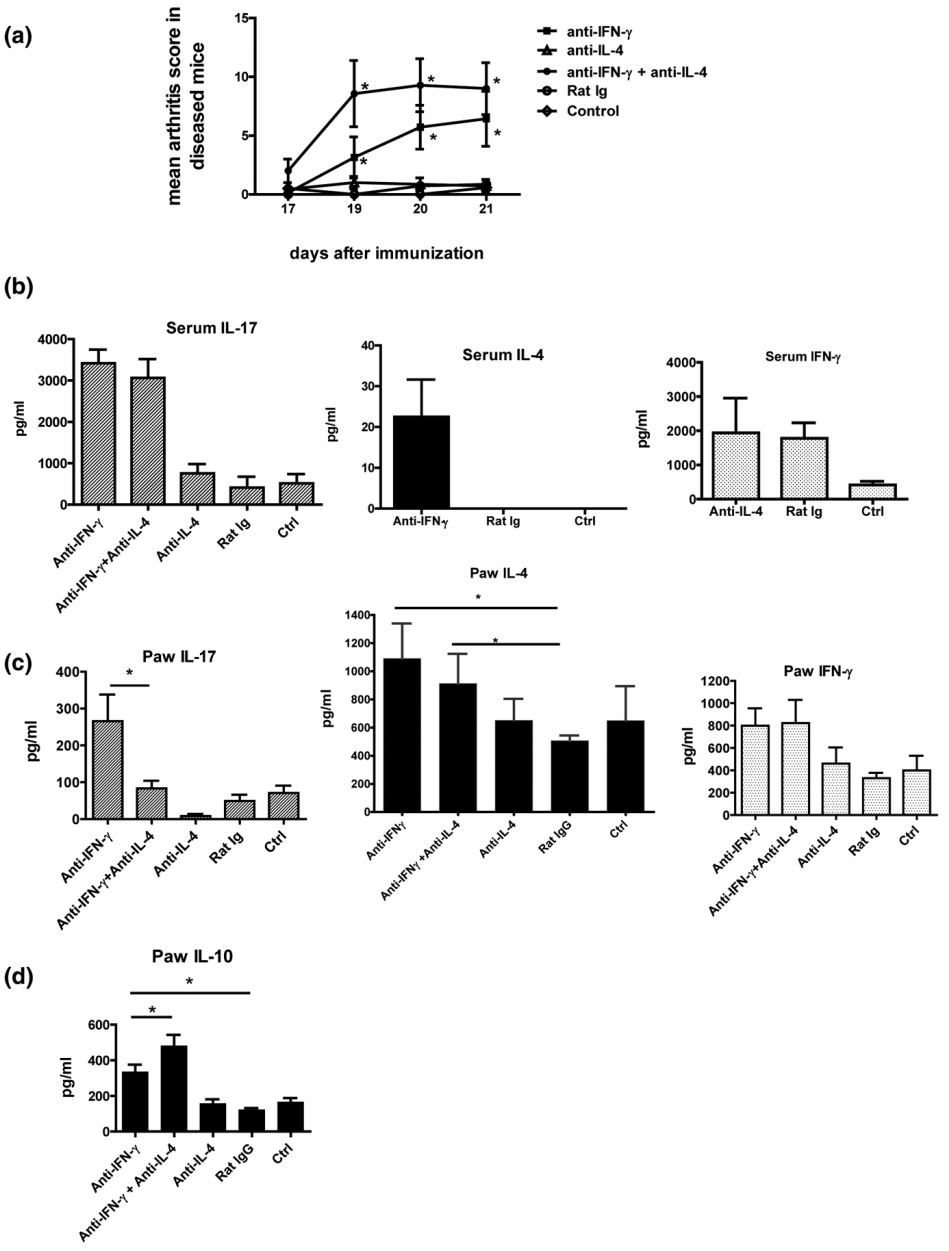
Regulation of IL-17 responses by Th2 cytokine IL-4 during the initiation phase of arthritis. **(a) **Neutralizing antibody to IFN-γ (clone R46A2, 100 μg/mouse/day), and/or neutralizing antibody to IL-4 (clone 11B11, 100 μg/mouse/day) was administered intraperitoneally from day 10 to 20 after immunization with collagen and complete Freund's adjuvant (CFA). Rat IgG was used as isotype control. The control group did not receive any antibody. Arthritis was assessed from day 10 by clinical scoring. Data is representative of two experiments, with n = 8 in each group. * *P *< 0.05. **(b) **Sera from day 22 after collagen immunization were analyzed for IL-17, IL-4, and IFN-γ by ELISA. Data is presented as mean +/- standard error of the mean (SEM). Data is representative of two experiments, with n = 8 in each group. **(c) **The paws of mice from the different groups were cultured in media overnight. The supernatants were analyzed for IL-17, IL-4 and IFN-γ by ELISA. Data presented as mean +/- SEM. Data is representative of two experiments, with n = 8 in each group.* *P *< 0.05. **(d) **The paws of mice from the different groups were cultured in media overnight. The supernatants were analyzed for IL-10 by ELISA. Data presented as mean +/- SEM. * *P *< 0.01.

Anti-IFN-γ or anti-IL-4 antibodies were administered, either alone or in combination, from day 10 to 20 after immunization with collagen and CFA. Mice that received neutralizing antibodies to IFN-γ alone developed an accelerated course of arthritis (Figure 3a), and the group that received neutralizing antibodies to both IL-4 and IFN-γ had significantly more severe arthritis than the anti-IFN-γ alone group. Furthermore, the onset and progression of arthritis in the anti-IFN-γ + anti-IL-4 group was faster than in the anti-IFN-γ group and reached a plateau by day 18, when the arthritis score in the anti-IFN-γ group was still increasing. Mice in which only IL-4 was neutralized did not have an accelerated course of arthritis, consistent with previous studies [31,32]. These results suggest that IFN-γ plays a more prominent protective role in CIA than IL-4, but that IL-4 plays a regulatory (and not a pathogenic) role in the absence of IFN-γ.

As both IFN-γ and IL-4 have been shown to suppress IL-17 production, it was possible that the increased severity of arthritis seen in the absence of IFN-γ and IL-4, in comparison to the absence of IFN-γ only, was secondary to higher levels of IL-17. Therefore serum levels of IL-17 were measured on day 22, immediately after completion of the 10-day course of neutralizing antibody administration (Figure 3b). Compared with mice that received neutralizing antibody to IFN-γ only, mice in which both IFN-γ and IL-4 were neutralized did not show a further elevation of IL-17 (Figure 3b). Thus the increased incidence of arthritis in the absence of IFN-γ and IL-4, versus IFN-γ only, does not correlate with further systemic elevation of IL-17. Additionally, while neutralization of IFN-γ resulted in elevation of serum IL-4, neutralization of endogenous IL-4 did not result in any change in serum IFN-γ levels (Figure 3b). Consistent with our previous findings, augmented IL-17 responses were not seen in collagen re-stimulation cultures of spleen and draining lymph nodes [Figure S2 in Additional data file 1] (data not shown). Thus the mechanisms underlying the protective role of IFN-γ seem to differ from the mechanisms mediating the protective role of IL-4, and the increase in disease induced following treatment with anti-IL-4 may be dependent on a mechanism distinct from IL-17.

IFN-γ, IL-4, and IL-17 levels in the target organs were measured by ELISA in supernatants of overnight paw cultures from the different groups of mice. Consistent with our previous findings, IFN-γ, IL-4, and IL-17 were elevated in the paws of arthritic mice from the anti-IFN-γ group (Figure 3c). Interestingly, the mice that received anti-IFN-γ + anti-IL-4 had similar levels of IFN-γ and IL-4 but lower levels of IL-17 in their paws even though they had more severe arthritis, suggesting that joints may be more sensitive to Th17-mediated inflammation in the absence of systemic protective Th1 and Th2 responses. The anti-IL-4 only group did not have elevated levels of IL-17 in their paws.

Although IL-4 has been shown to down-regulate IL-17 production *in vitro *[23], a similar effect of endogenous IL-4 was not seen *in vivo*, because neither neutralizing antibodies to IL-4 alone or IL-4 in combination with IFN-γ resulted in incremental elevation of serum IL-17 levels (Figure 3b), IL-17 production in cultured spleen or lymph node cells [Figure S2 in Additional data file 1] (data not shown), or IL-17 production in the joints (Figure 3c). Thus, endogenous IL-4 does not play a major role in the regulation of endogenous IL-17 in the early phase of CIA. It is possible that IL-4 exerts a protective function in the absence of IFN-γ by some other mechanism, possibly by inducing other cytokines such as IL-10, and/or by direct effects on synovial cells.

As IL-10 has been found to be associated with a less pathogenic phenotype of Th17 cells in the mouse model of multiple sclerosis [33], we evaluated IL-10 responses in mice that received neutralizing antibodies to IL-4 and/or IFN-γ. Arthritic paws from mice that received neutralizing antibodies to IFN-γ or IL-4 + IFN-γ had increased levels of IL-10 (Figure 3d). IL-10 was not detectable in collagen re-challenge cultures of lymphoid organs (data not shown). This suggests that in CIA, endogenous regulatory effects of IL-4 are not mediated through systemic production of IL-10. The elevated levels of IL-10 in the arthritic joints could in part reflect IL-10 production by synovial cells.

**Figure 4:**
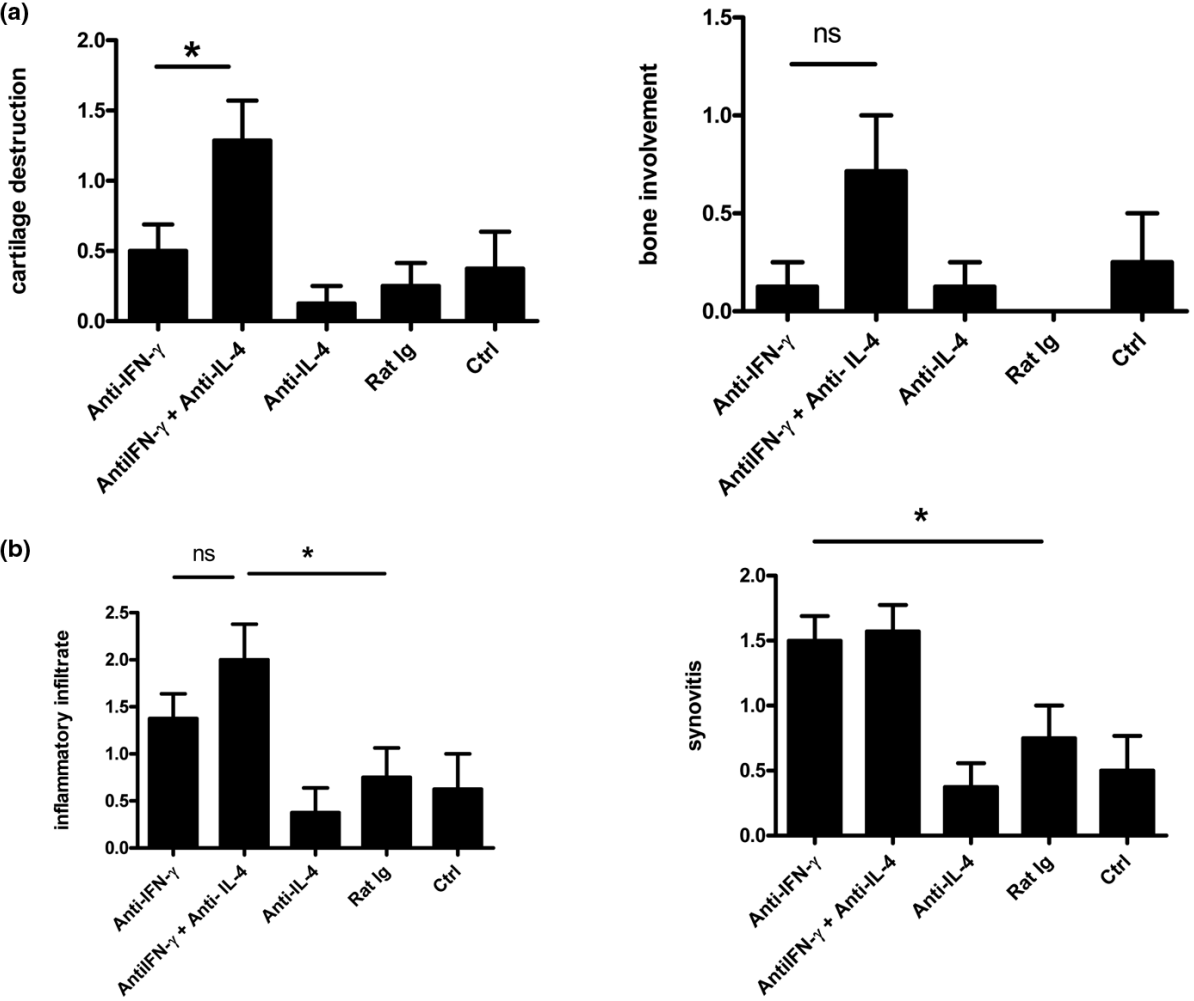
Degree of tissue inflammation and bone and cartilage destruction in the absence of endogenous Th1 and Th2 cytokines. **(a and b) **Tissue sections of arthritic hind paw from mice that received neutralizing antibodies to IFN-γ alone, IFN-γ + IL-4, IL-4 alone, rat IgG or no antibody were stained with hematoxylin and eosin. The sections were scored for inflammatory infiltrate, synovitis, bone damage and cartilage destruction. Data are presented as mean +/- standard deviation (SD). * *P *< 0.05.

**Figure 5:**
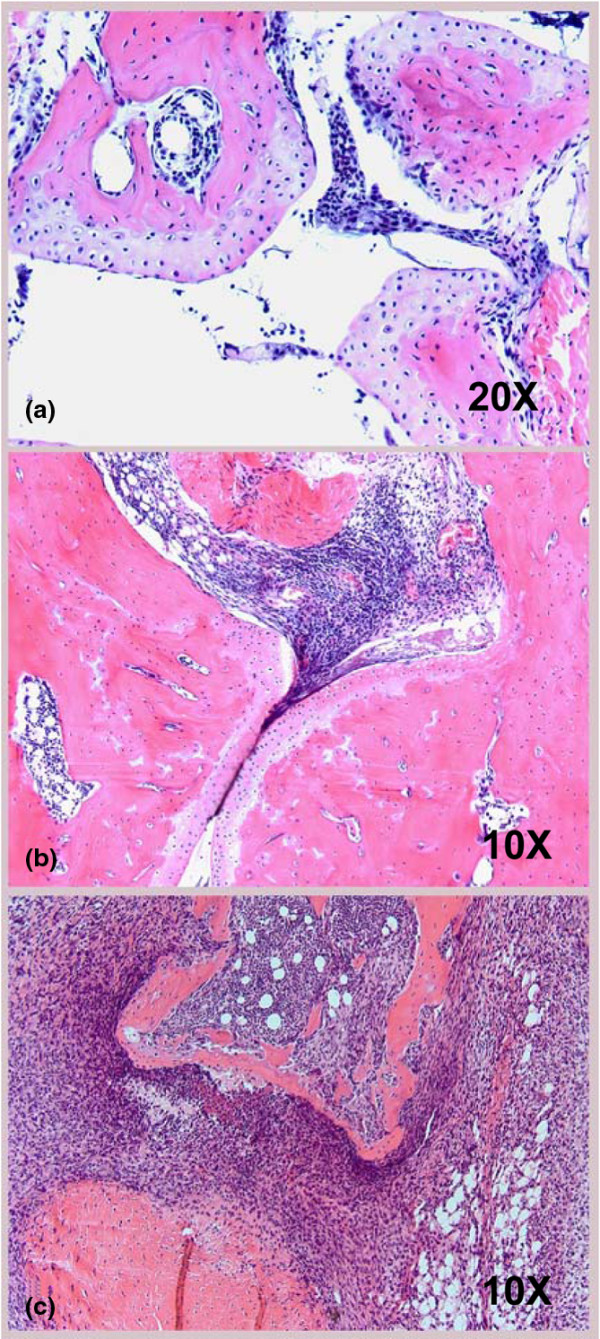
Hematoxylin and eosin staining of paws from mice that received various treatments. **(a) **Ankle joint (clinical score 0) from a mouse that received rat IgG control antibody demonstrates a mild inflammatory infiltrate within a non-distorted joint space. A mild degree of synovial hyperplasia is also present. No significant cartilage or bone destruction is seen (hematoxylin and eosin, 20 ×). **(b) **Arthritic joint (clinical score 4) from a mouse that received anti-IFN-γ antibody, demonstrating inflammatory cells, with partial filling of the joint space. Mild synovial hyperplasia is present along with early, minimal alteration of cartilage. Inflammatory changes extend into the adjacent soft tissue. No significant bony changes are present and the joint space is otherwise intact (hematoxylin and eosin, 10 ×). **(c) **Arthritic joint (clinical score 4) from a mouse that received neutralizing antibodies to IFN-γ + IL-4, demonstrating severe inflammatory changes including complete filling of the joint space and extension to the soft tissue. Cartilage is significantly destroyed and the bone shows a substantial amount of destruction and remodeling (hematoxylin and eosin, 10 ×).

Previous studies have shown that administration of IL-4 can protect against bone damage in CIA [20,22], and IL-4 is known to have direct inhibitory effects on osteoclastogenesis distinct from its effects on T and B cells [22]. Interestingly, we found that the increased severity of arthritis seen in the absence of IFN-γ and IL-4 was associated with increased bone and cartilage damage as compared with the anti-IFN-γ only group, despite the fact that both groups showed a similar degree of synovitis and inflammatory infiltrate (Figures 4a and 4b). The anti-IL-4 only group did not show any increased bone or cartilage damage over baseline. Figures 5a to 5c illustrate the degree of inflammatory infiltrate and bone and cartilage damage associated with the neutralization of IFN-γ compared with IFN-γ + IL-4.

### Relative contribution of IFN-γ and IL-4 in the regulation of IL-17 *in vivo*

**Figure 6:**
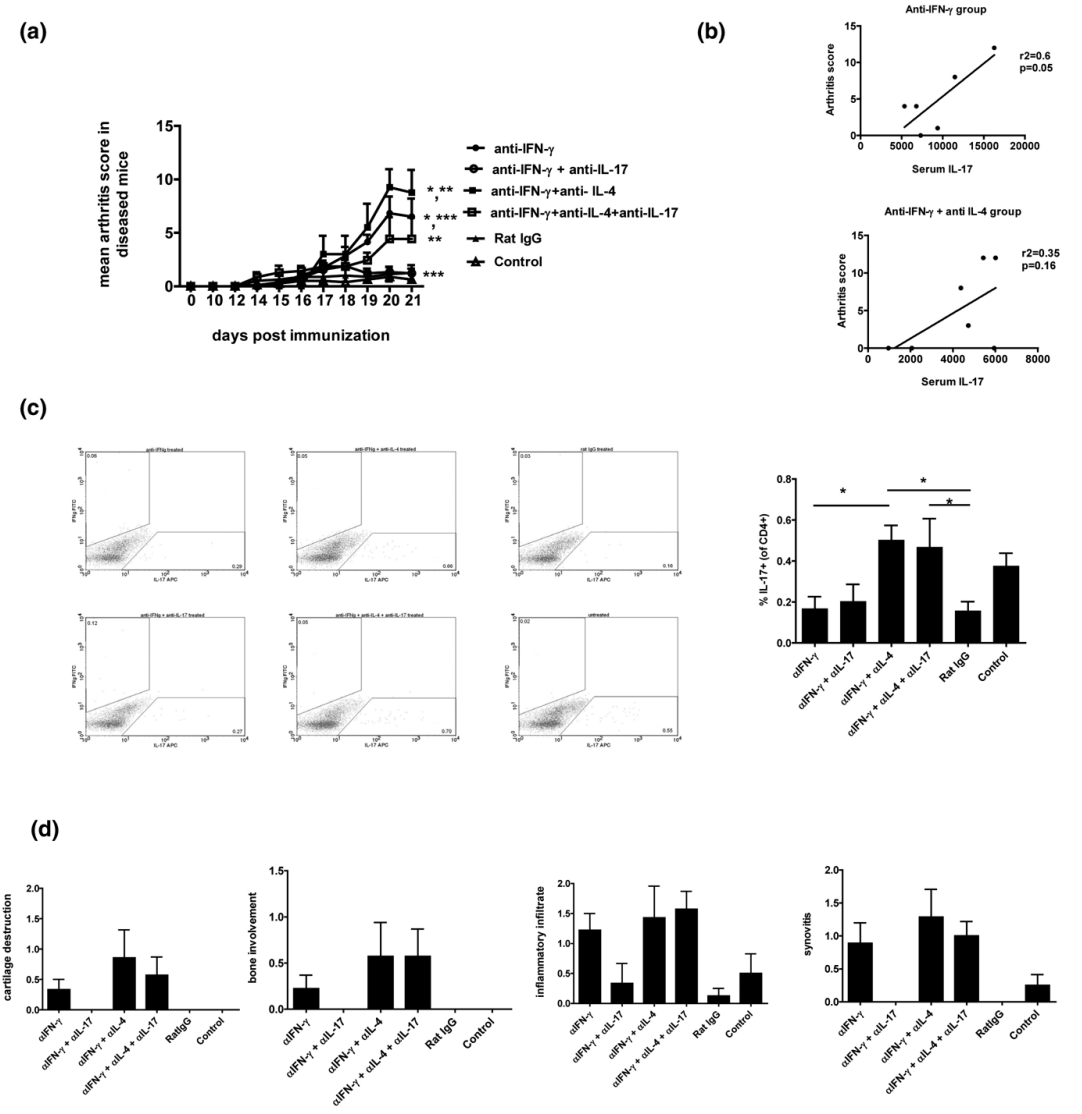
Effect of neutralization of IL-17 in the presence or absence of anti-IFN-γ and/or anti-IL-4 during the initiation phase of arthritis. **(a) **Neutralizing antibody to IFN-γ (clone R46A2, 100 μg/mouse/day), and/or neutralizing antibody to IL-4 (clone 11B11, 100 μg/mouse/day), and neutralizing antibody to IL-17 (clone M210, 100 μg/mouse/day) was administered intraperitoneally from day 10 to 20 after immunization with collagen and complete Freund's adjuvant (CFA). Rat IgG was used as isotype control. The control group did not receive any antibody. Arthritis was assessed from day 10 by clinical scoring. n = 8 to 9/group. * *P *< 0.05, ** *P *< 0.01 *** *P *< 0.001. **(b) **Correlation analysis of serum IL-17 to clinical scores of arthritis severity in the anti-IFN-γ and the anti-IFN-γ + anti-IL-4 groups. **(c) **Splenocytes from the different groups were cultured overnight with collagen. Following six-hour stimulation with PMA/ionomycin/BrefeldinA, cells were stained with fluorescent labeled anti-CD4, anti-IL-17, anti-IFN-γ, and anti-IL-4 antibodies and analyzed on FACS Calibur using Cell Quest software. * *P *< 0.05. **(d) **Tissue sections of arthritic hind paw from mice that received neutralizing antibodies to IFN-γ alone, IFN-γ + IL-17, IFN-γ + IL-4, IFN-γ + IL-4 + IL-17, rat IgG or no antibody were stained with hematoxylin and eosin. The sections were scored for inflammatory infiltrate, synovitis, bone damage and cartilage destruction. Data are presented as mean +/- standard deviation.

As the administration of neutralizing antibody to IFN-γ with or without anti-IL-4 antibody was associated with differential regulation of IL-17 responses *in vivo*, we wanted to confirm and evaluate the role of IL-17 in mediating joint inflammation by administering neutralizing antibody to IL-17 along with anti-IFN-γ and/or anti-IL-4 antibodies. Anti-IL-17 antibody was administered in combination with anti-IFN-γ antibody or anti-IFN-γ + anti-IL-4 antibodies from day 10 to 20 after immunization with collagen and CFA (Figure 6a). Consistent with our previous findings, mice that received anti-IFN-γ + anti-IL-4 antibodies had more severe arthritis than the anti-IFN-γ alone group. Interestingly, neutralizing antibody to IL-17 completely abrogated disease in the anti-IFN-γ alone group, whereas anti-IL-17 had only a partial effect on arthritis in the mice that received anti-IFN-γ + anti-IL-4 (Figure 6a). These results suggest that treatment with neutralizing antibodies to IFN-γ and IL-4 prompts the development of joint inflammation that is in part independent of systemic IL-17. In order to further elucidate the relative contribution of IL-17 to disease in mice receiving anti-IFN-γ versus anti-IFN-γ + anti-IL-4, we analyzed the correlation between serum IL-17 and arthritis severity (Figure 6b). Although the absolute levels of serum IL-17 were comparable between the groups, there was a significant correlation between IL-17 and arthritis severity in the anti-IFN-γ group but not in the anti-IFN-γ + anti-IL-4 group (we could not address the IL-17/IFN-γ ratio in these mice, as treatment with anti-IFN-γ precludes ELISA for IFN-γ in the serum). The expression of IFN-γ, IL-4, and IL-17 in the paws, as well as IL-17 production by spleen and lymph node cultures, was consistent with previous experiments (data not shown).

Neutralization of IFN-γ *in vivo *is associated with elevated serum IL-17, with no further up-regulation in IL-17 with combined neutralization of IFN-γ and IL-4 (Figure 3b). Previously reported studies have consistently shown that neutralization of IFN-γ, as well as IL-4, is required for the optimal differentiation of Th17 cells *in vitro*. Furthermore, some studies, mostly in humans, have reported the presence of Th17 cells expressing both IFN-γ and IL-17. Hence we wanted to investigate whether the IL-17 and IFN-γ responses in our experiments were associated with dual positive Th1/Th17 cells and/or if the increase in the systemic IL-17 responses was associated with an increase in the generation of Th17 cells *in vivo*. Splenocytes from the various groups were analyzed for IL-17, IFN-γ, and or IL-4 production by intracellular flow cytometry. Our data shows that IFN-γ and IL-17 were produced by a discrete population of T cells and neutralization of both IFN-γ and IL-4 was associated with increased differentiation of Th17 cells *in vivo *(Figure 6c). IL-4-producing T cells were not detectable. Surprisingly, neutralization of IFN-γ alone was not associated with an increase in the number of Th17 cells, even though this group of mice had the highest levels of serum IL-17 (Figures 3b and 6c). On the other hand, neutralization of both IFN-γ + IL-4 was associated with an increase in the number of Th17 cells, but lower amounts of serum and paw IL-17 than the anti-IFN-γ alone group (Figures 3b and 3c and 6c). Thus, Th17 differentiation and IL-17 production may be differentially regulated *in vivo*, with IFN-γ primarily suppressing IL-17 production and IL-4 primarily suppressing Th17 differentiation. Alternatively, the Th17 cells that differentiate in the presence of anti-IFN-γ and anti-IL-4 may be differently activated, resulting in a more pathogenic phenotype despite reduced IL-17 production.

Tissue sections of inflamed joints were stained with H&E and analyzed for inflammatory infiltrate, synovitis, cartilage destruction, and bone erosion. Consistent with arthritis severity scores, neutralizing antibody to IL-17 completely protected the joints of mice treated with anti-IFN-γ but had no effect on the severe joint inflammation and destruction observed in mice treated with anti-IFN-γ and anti-IL-4 (Figure 6D). These results further support the supposition that the increase in disease seen in the presence of anti-IFN-γ + anti-IL-4 is mediated by a mechanism distinct from systemic production of IL-17.

## Discussion

In the past few years the pathogenic role of IFN-γ in immune-mediated diseases such as RA and CIA has been called into question. Several studies have shown increased IL-17, as well as inflammatory mediators induced by IL-17, in the RA synovium [2,4-6,34-38]. IL-6 + transforming growth factor (TGF)-β, IL-21 and IL-23 are important in the generation, expansion and maintenance of Th17 cells [28,39-42]. All of these Th17-associated cytokines are found in RA synovial tissue. In addition, IL-17 can synergize with TNF-α and IL-1, two cytokines that are known to play an important role in the pathogenesis of RA. Thus there is considerable interest in new strategies to inhibit Th17 cells, and the safest approach is likely to be one that restores the immune homeostasis and T cell subset balance, thereby minimizing autoimmune inflammation without crippling anti-microbial and anti-tumor responses.

IFN-γ and IL-4 suppress Th17 differentiation *in vitro*, as well as secretion of IL-17 from committed Th17 cells. Thus IFN-γ and IL-4 represent endogenous mechanisms for regulating Th17-mediated inflammation, which may play a role in preventing or controlling autoimmunity [11,12,23]. In fact, knocking out IFN-γ worsens CIA and renders resistant strains of mice susceptible to disease, which is associated with increased IL-17 production [25,26]. Furthermore, we have found that treatment with exogenous IL-4, in the form of DCs genetically engineered to produce IL-4, suppresses IL-17 and reduces the incidence and severity of CIA [21,23].

Although there have been significant advances in understanding the development and maintenance of Th17 cells *in vitro*, the endogenous regulation of Th17 responses during the development of arthritis is still under investigation. Various antigenic stimuli can trigger IL-17 responses *in vivo *and not all of them will result in systemic or organ specific autoimmunity in animal models, implying that endogenous regulation of IL-17 responses is important in the prevention or attenuation of autoimmunity. Herein we present data on the regulation of IL-17 responses by the Th1 cytokine IFN-γ and the Th2 cytokine IL-4 in CIA.

Our studies show that the level of systemic IL-17 is not directly associated with arthritis, but the ratio of systemic IL-17/IFN-γ is an important predictor of target organ damage (Figures 1a to 1c). These results suggest that disease outcome is not determined solely by the absolute level of the pathogenic cytokine, but rather by the balance between pathogenic and protective signals. How these competing signals regulate disease pathogenesis at the molecular level is not clear. One possibility is that these signals modulate trafficking of Th17 cells to the joint, either by altering expression of chemokines by cells of the synovium or expression of chemokine receptors by T cells. Once in the joint, Th17 cells can then induce inflammation and recruitment of other inflammatory cells. Interestingly, after immunization similar high levels of IL-17 were detectable in the serum of both arthritic and non-arthritic animals, but IL-17 was only found in arthritic joints (Figures 1a and 1d). Non-arthritic paws from arthritic mice or paws from non-arthritic mice do not have detectable IL-17. In addition, arthritic joints had higher levels of IFN-γ, and IL-4 than non-arthritic joints, suggesting that once target organ inflammation is initiated there is recruitment of both inflammatory and anti-inflammatory cell types. Further studies are needed to determine the effect of the systemic Th1/Th17 balance on T cell homing and recruitment to the joint.

Our results implied that the balance between Th1 and Th17 cells played an important role in disease outcome, so neutralizing antibody to IFN-γ was administered to perturb this balance. Consistent with previous data suggesting that IFN-γ negatively regulates IL-17 responses and clinical arthritis, mice that had received anti-IFN-γ antibody had accelerated arthritis associated with elevated levels of IL-17 (Figures 2a and 2b). The arthritic paws from these mice had elevated levels of IFN-γ, IL-4, and IL-17 (Figure 2c). A similar elevation of IFN-γ, IL-4, and IL-17 was seen in arthritic joints in CIA mice not treated with cytokine-neutralizing antibodies (Figure 1d). This indicates that the systemic cytokine response is distinct from that in the target organ and the balance of Th1/Th17 response is critical in the systemic immune events leading up to target organ damage. Once the target organ damage is initiated, the IFN-γ, IL-4, and IL-17 responses within the arthritic joint, in the presence or absence of anti-IFN-γ, are similar. This is consistent with a recent study demonstrating that Th17 cells were predominant in the draining inguinal lymph nodes whereas IL-17-producing γδ T cells were predominant in the inflamed joint [27].

Although increased systemic IL-17 production was associated with accelerated disease, the increase in systemic IL-4 (Figure 2b) was potentially surprising, in view of previous reports of the suppressive effects of IL-4-based therapies on arthritis and the known ability of IL-4 to suppress IL-17 responses *in vitro*. However, the appearance of IL-4 in this situation could represent a back-up mechanism for immune regulation that was able to emerge only with neutralization of IFN-γ. To evaluate the protective or pathogenic role of IL-4 in accelerated CIA, neutralizing anti-IL-4 antibody was administered in conjunction with neutralizing anti-IFN-γ antibody. This resulted in significantly increased severity and accelerated onset of arthritis over mice that received neutralizing antibodies to IFN-γ alone (Figure 3a). Consistent with previous reports, mice that received neutralizing antibody to IL-4 alone did not have an accelerated course of arthritis [32]. Thus endogenous IFN-γ seems to play a more prominent role than IL-4 in down-regulating arthritis. This could be due to the fact that the immune response to collagen in DBA mice is primarily Th1 and Th17, with undetectable levels of IL-4 in the serum or supernatants of collagen-stimulated lymphoid organs (data not shown). However, because IFN-γ is a potent suppressor of Th2 as well as Th17 development, neutralizing IFN-γ allows for the unmasking of the Th2 response. Therefore, the experiments involving the administration of neutralizing antibodies to both IFN-γ and IL-4 suggest a secondary protective role of endogenous IL-4 in CIA.

As both IFN-γ and IL-4 suppress IL-17 *in vitro*, one would expect that the increased severity of arthritis seen with anti-IFN-γ + anti-IL-4 would be associated with increased IL-17. However, there was no further increase in the serum levels of IL-17 in the anti-IFN-γ + anti-IL-4 groups, in comparison to the anti-IFN-γ group (Figure 3b). In addition, the arthritis in the anti-IFN-γ group was associated with significantly elevated IFN-γ, IL-4, and IL-17 levels in the joints, whereas the arthritis in the anti-IFN-γ + anti-IL-4 group was associated with significant increase in IFN-γ and IL-4 and a modest increase in IL-17 responses (Figure 3c).

Th17 cells that did not co-express IL-10 were found to have a higher pathogenic potential in the mouse model of multiple sclerosis than Th17 cells that expressed IL-10 [33]. It is possible that the increased arthritis in the presence of anti-IFN-γ + anti IL-4 is associated with the generation of a more aggressive phenotype of Th17 cells, one that may be associated with reduced levels of IL-10. However, IL-10 levels were higher in the paws of mice that received anti-IFN-γ + anti-IL-4 than in paws of mice that received anti-IFN-γ alone (Figure 3d). This would suggest that the phenotype of Th17 responses in CIA in the absence of IL-4 is independent of IL-10.

As mentioned earlier, IL-4 possibly exerts a protective role in CIA through effects on cartilage and bone. There was a similar degree of inflammatory infiltrate and synovitis in the absence of IFN-γ alone or in the absence of IFN-γ + IL-4 (Figures 4 and 5). However, there was more bone and cartilage destruction in the absence of IFN-γ + IL-4, suggesting a more aggressive phenotype of IL-17 responses in the absence of both Th1 and Th2 responses (Figures 4 and 5). It is also possible that IL-4 could have direct protective effects on bone and cartilage damage, independent of regulation of IL-17. Intra-articular delivery of adenoviral vector associated Th2 cytokines, IL-4 and IL-13, has been shown to slow bone and cartilage damage in rat adjuvant arthritis [43,44]. In addition, IL-4 can directly down-regulate osteoclastogenesis through inhibition of RANKL activity [45,46]. Interestingly, in patients with RA a polymorphism in the IL-4 receptor that results in reduced responsiveness to IL-4 is associated with rapidly erosive disease, suggesting that IL-4 plays a protective role in RA [47].

The role of endogenous IFN-γ and IL-4 in the differential regulation of IL-17, leading up to arthritis, was evaluated in experiments involving administration of anti-IL-17 antibody along with anti-IFN-γ or anti-IFN-γ + anti-IL-4 antibodies. Administration of anti-IL-17 antibody completely abrogated the arthritis associated with anti-IFN-γ alone (Figures 6a and 6d). In contrast, the augmented arthritis with anti-IFN-γ + anti-IL-4 antibodies was only partially suppressed with anti-IL-17 antibody (Figures 6a and 6d). Interestingly, although the anti-IFN-γ group had similar levels of serum and elevated levels of paw IL-17 in comparison to the anti-IFN-γ + anti-IL-4 groups (Figures 3b and 3c) and the arthritis was completely dependent on IL-17, the numbers of Th17 cells *in vivo *were not increased (Figures 6a, c and 6d). This suggests that IFN-γ plays a major role in the regulation of IL-17 secretion and has only a modest effect on the number of Th17 cells *in vivo*. The anti-IFN-γ + anti-IL-4 group had an elevated number of Th17 cells *in vivo *(Figure 6c) and yet did not have increased levels of serum and paw IL-17 levels (Figures 3c and 3d), suggesting that the Th17 cells generated under this condition produced less IL-17 on a per cell basis.

Further, the neutralization of endogenous IFN-γ or IFN-γ + IL-4 each lead to joint inflammation by distinct pathways, one completely dependent on IL-17 and the other only partially mediated by IL-17 (Figure 6a). The cytokine responses within the arthritic joints are also different: anti-IFN-γ is associated with elevated IL-17, whereas anti-IFN-γ + anti-IL-4 is associated with a less striking elevation of IL-17 (Figure 3c). These data provide insight into the heterogeneity of systemic as well as joint-specific immune events underlying inflammatory arthritis.

Another intriguing finding was the significant level of IL-4 in the arthritic joints (Figures 1d, 2c and 3c), in the absence of detectable amounts of IL-4 in the lymphoid organs and only small amounts of IL-4 in the serum (Figures 2b and 3b). The levels of IL-4 correlated with levels of IL-17 in the joints (Figure 1e). It is possible that the source of IL-4 in the sera and the joints may be different - T cells in the peripheral circulation versus mast cells in arthritic joints. Whatever the source of IL-4 in the joints, our results suggest that its role in CIA is primarily regulatory rather than pathogenic, at least in the absence of IFN-γ.

## Conclusions

We conclude that the absolute magnitude of the IL-17 response is not the sole initiator of autoimmunity, but that the Th1/Th2/Th17 balance is of primary importance. It is plausible that the absence of endogenous IL-4 and IFN-γ generates a much more aggressive phenotype of Th17 cells than absence of IFN-γ alone. We show that the absolute quantity of IL-17 as well as the consequences of this IL-17 response are regulated differentially by endogenous Th1 and Th2 cytokines in CIA. IFN-γ is the primary suppressor of endogenous IL-17, while the role of IL-4 is unmasked only in the absence of IFN-γ. IL-4 and IFN-γ can play protective roles in CIA via discrete mechanisms; IFN-γ inhibits disease primarily through suppression of IL-17, and IL-4 acts by altering the cytokine milieu, phenotype of Th17 cells, and/or by directly inhibiting bone and cartilage damage.

## Abbreviations

CFA: complete Freund's adjuvant; CIA: collagen-induced arthritis; DC: dendritic cell; ELISA: enzyme linked immunosorbant assay; H&E: hematoxylin and eosin; IFN: interferon; IL: interleukin; OA: osteoarthritis; RA: rheumatoid arthritis; RANKL: receptor activator of NF-kB ligand; TGF: transforming growth factor; TNF: tumor necrosis factor.

## Competing interests

The authors declare that they have no competing interests.

## Authors' contributions

SS and LAC performed the experiments, analyzed data and prepared the manuscript; PW, DBD and JE provided technical help with the experiments; JMJ and MJW performed the scoring of histopathology slides; and DAF reviewed experiment design, data and manuscript.

## Supplementary Material

Additional file 1 **Figure S1 **that shows the IL-17 responses in splenocytes and single cell suspensions of draining inguinal lymph nodes from anti-IFN-γ, rat IgG or control group in response to *in vitro *stimulation with collagen and **Figure S2 **that shows the IL-17 and IFN-γ responses in splenocytes from anti-IFN-γ, anti-IL-4, anti-IFN-γ + anti-IL-4, rat IgG or control groups in response to *in vitro *stimulation with collagen. Click here for file
