# Supplementary material for: Regulation of pathogenic IL-17 responses in collagen-induced arthritis: roles of endogenous interferon-gamma and IL-4
Source: Arthritis Res Ther. 2009 Oct 26;11(5):R158. doi: 10.1186/ar2838 (PMC2787258; doi:10.1186/ar2838)
Supplement: Additional file 1 — Figure S1 that shows the IL-17 responses in splenocytes and single cell suspensions of draining inguinal lymph nodes from anti-IFN-γ, rat IgG or control group in response to in vitro stimulation with collagen and Figure S2 that shows the IL-17 and IFN-γ responses in splenocytes from anti-IFN-γ, anti-IL-4, anti-IFN-γ + anti-IL-4, rat IgG or control groups in response to in vitro stimulation with collagen. [file ar2838-S1.ppt]

## Slide 1
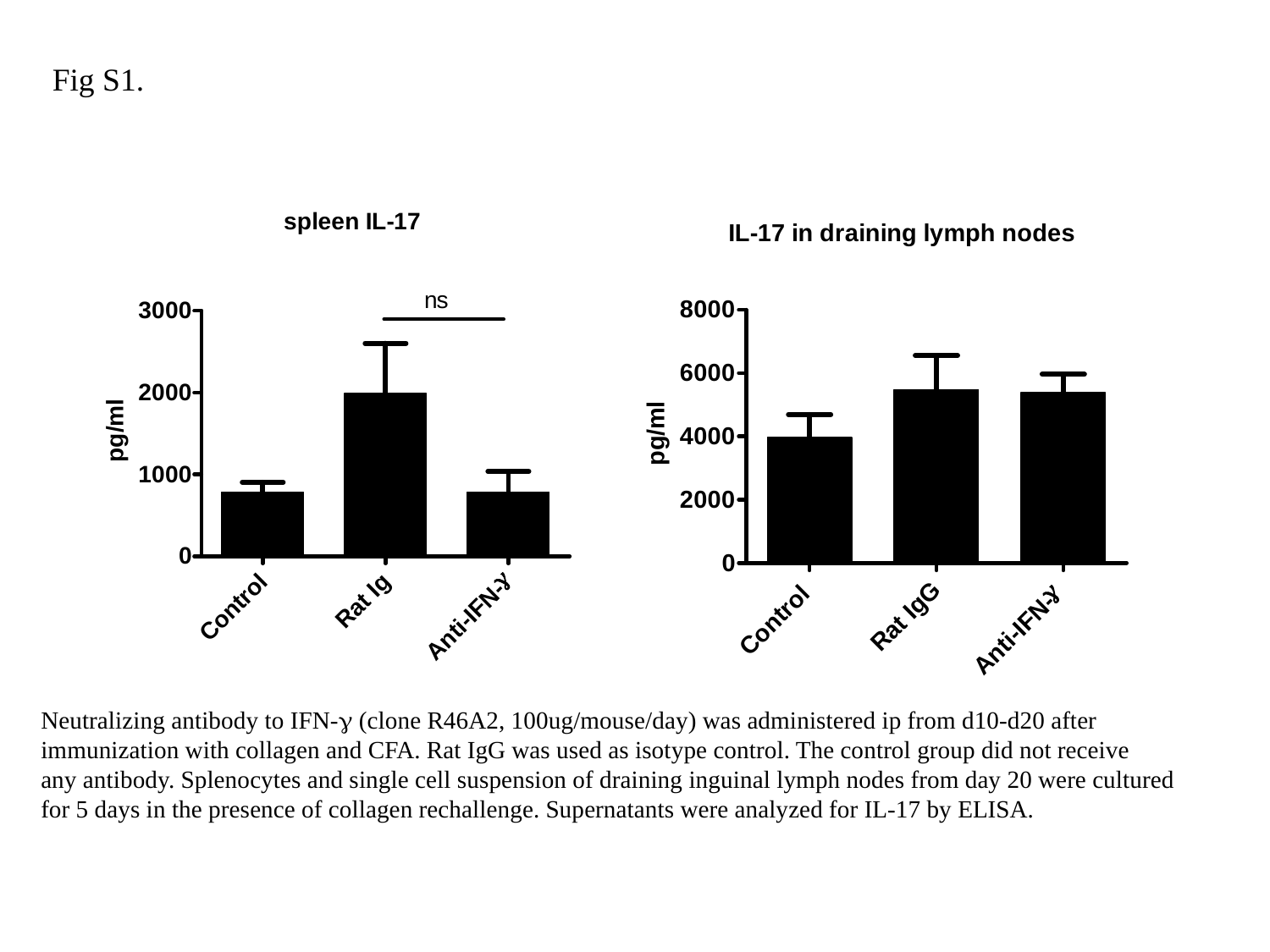

Fig S1.
Neutralizing antibody to IFN-γ (clone R46A2, 100ug/mouse/day) was administered ip from d10-d20 after
immunization with collagen and CFA. Rat IgG was used as isotype control. The control group did not receive
any antibody. Splenocytes and single cell suspension of draining inguinal lymph nodes from day 20 were cultured
for 5 days in the presence of collagen rechallenge. Supernatants were analyzed for IL-17 by ELISA.

## Slide 2
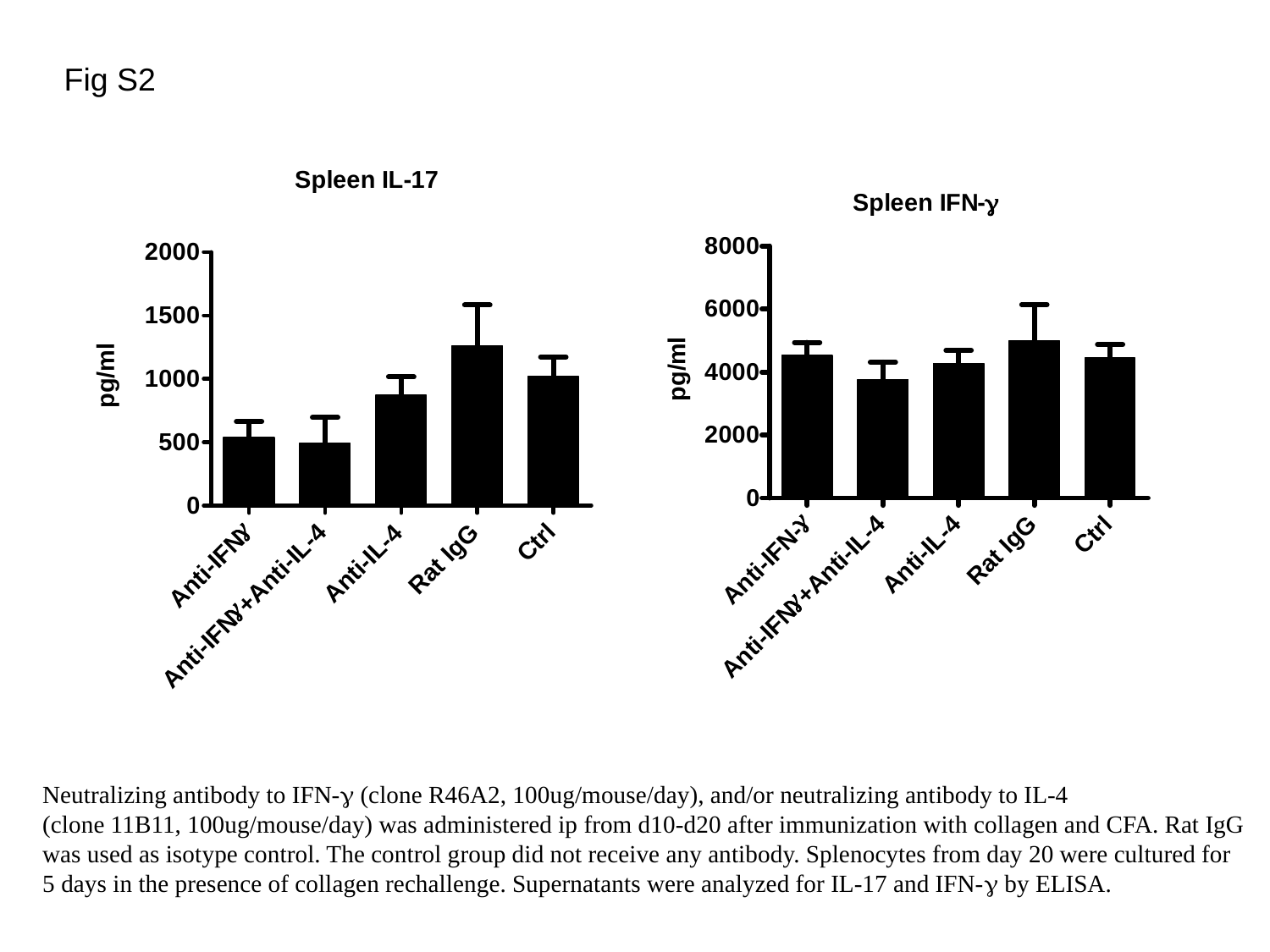

Fig S2
Neutralizing antibody to IFN-γ (clone R46A2, 100ug/mouse/day), and/or neutralizing antibody to IL-4
(clone 11B11, 100ug/mouse/day) was administered ip from d10-d20 after immunization with collagen and CFA. Rat IgG
was used as isotype control. The control group did not receive any antibody. Splenocytes from day 20 were cultured for
5 days in the presence of collagen rechallenge. Supernatants were analyzed for IL-17 and IFN-γ by ELISA.
